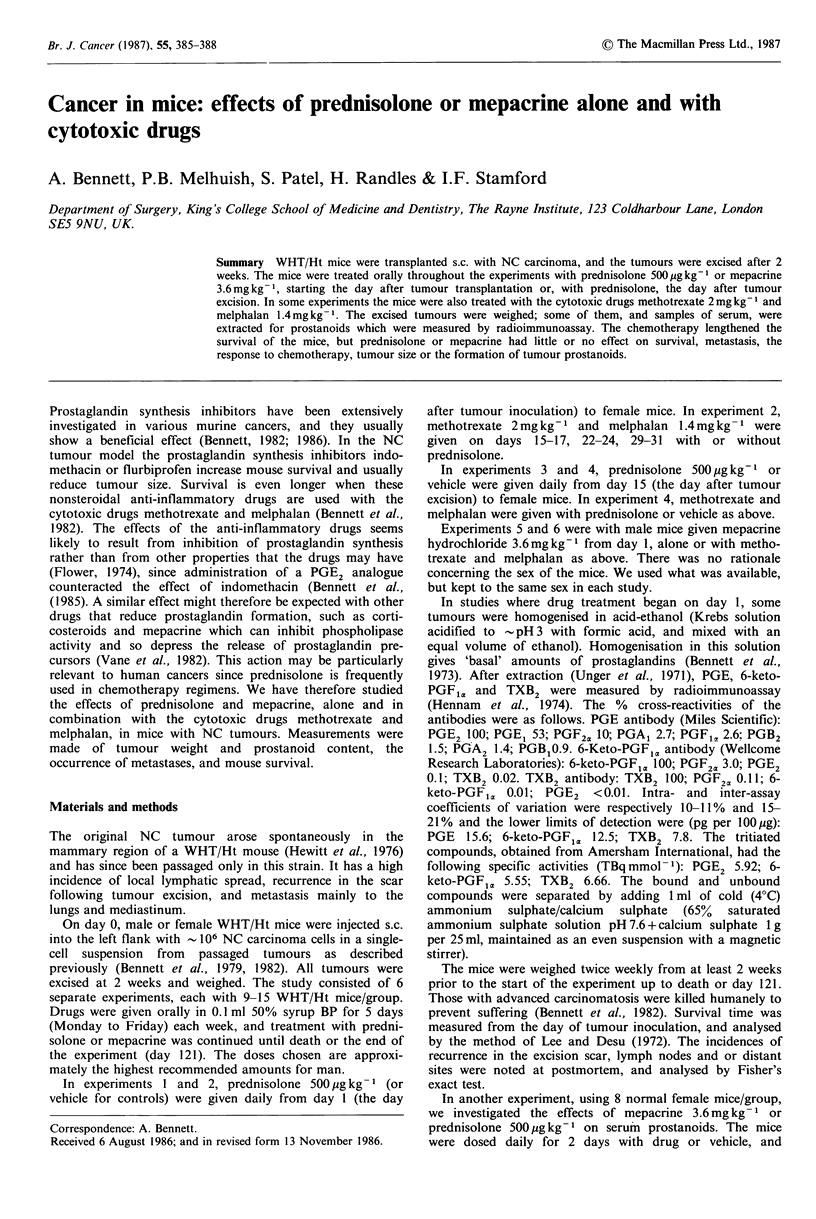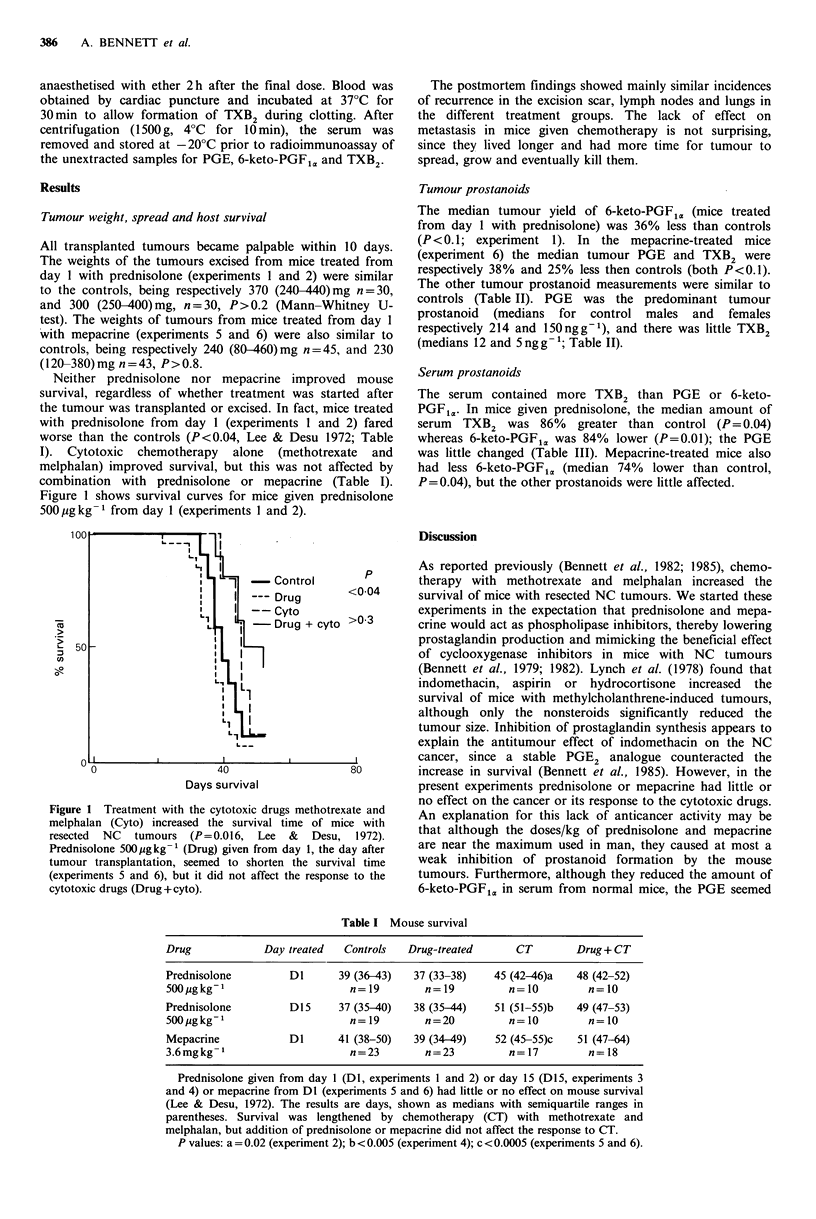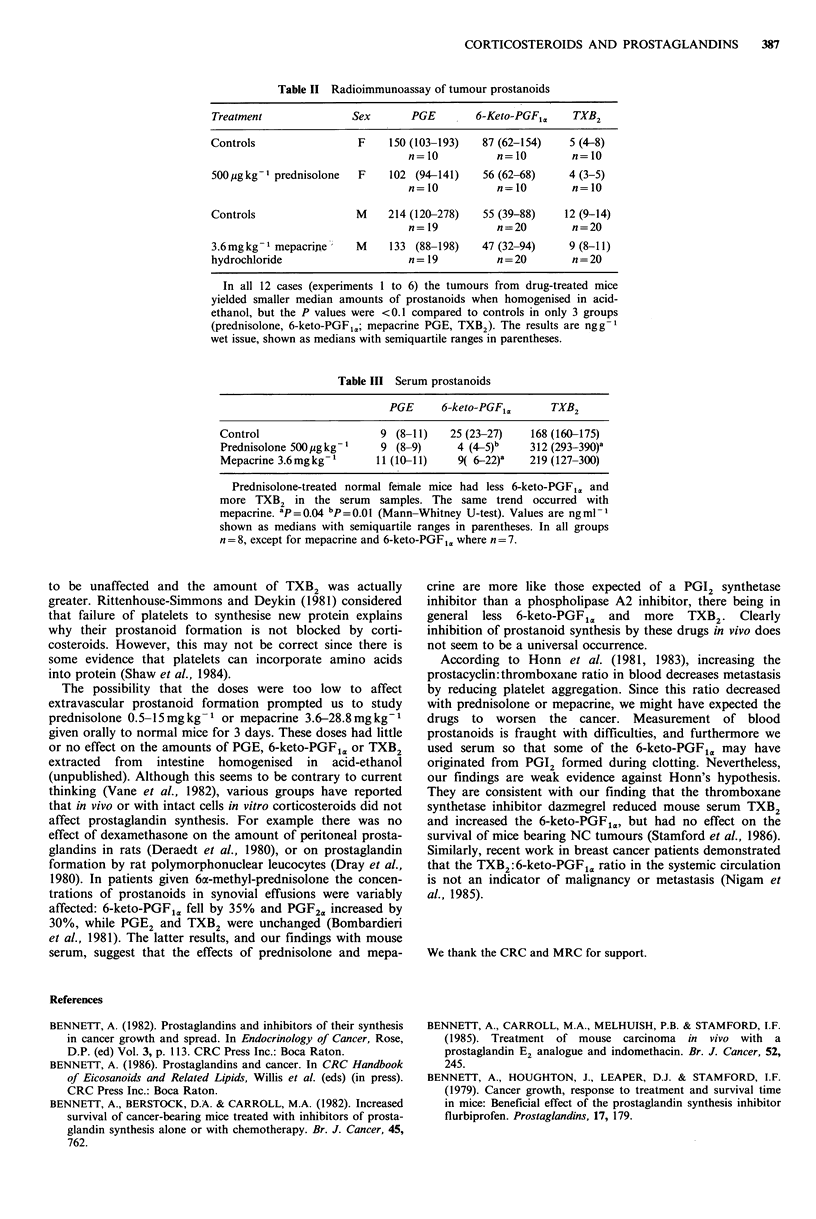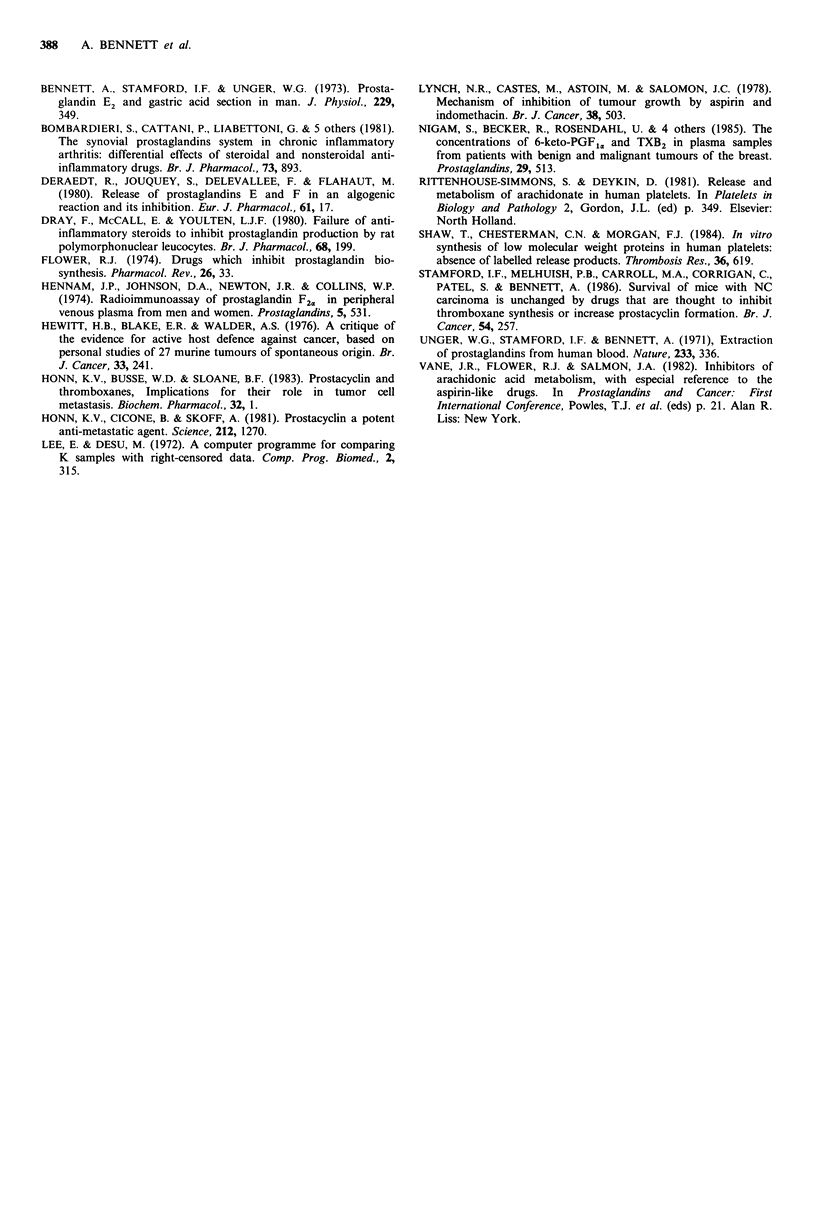# Cancer in mice: effects of prednisolone or mepacrine alone and with cytotoxic drugs.

**DOI:** 10.1038/bjc.1987.77

**Published:** 1987-04

**Authors:** A. Bennett, P. B. Melhuish, S. Patel, H. Randles, I. F. Stamford

## Abstract

WHT/Ht mice were transplanted s.c. with NC carcinoma, and the tumours were excised after 2 weeks. The mice were treated orally throughout the experiments with prednisolone 500 micrograms kg-1 or mepacrine 3.6 mg kg-1, starting the day after tumour transplantation or, with prednisolone, the day after tumour excision. In some experiments the mice were also treated with the cytotoxic drugs methotrexate 2 mg kg-1 and melphalan 1.4 mg kg-1. The excised tumours were weighed; some of them, and samples of serum, were extracted for prostanoids which were measured by radioimmunoassay. The chemotherapy lengthened the survival of the mice, but prednisolone or mepacrine had little or no effect on survival, metastasis, the response to chemotherapy, tumour size or the formation of tumour prostanoids.


*Br. J. Cancer* (1987), **55**, 385–388
© The Macmillan Press Ltd., 1987

### Cancer in mice: effects of prednisolone or mepacrine alone and with cytotoxic drugs

A. Bennett, P.B. Melhuish, S. Patel, H. Randles & I.F. Stamford

*Department of Surgery, King's College School of Medicine and Dentistry, The Rayne Institute, 123 Coldharbour Lane, London SE5 9NU, UK.*

**Summary** WHT/Ht mice were transplanted s.c. with NC carcinoma, and the tumours were excised after 2 weeks. The mice were treated orally throughout the experiments with prednisolone 500 $\mu g\,kg^{-1}$ or mepacrine 3.6 $mg\,kg^{-1}$, starting the day after tumour transplantation or, with prednisolone, the day after tumour excision. In some experiments the mice were also treated with the cytotoxic drugs methotrexate 2 $mg\,kg^{-1}$ and melphalan 1.4 $mg\,kg^{-1}$. The excised tumours were weighed; some of them, and samples of serum, were extracted for prostanoids which were measured by radioimmunoassay. The chemotherapy lengthened the survival of the mice, but prednisolone or mepacrine had little or no effect on survival, metastasis, the response to chemotherapy, tumour size or the formation of tumour prostanoids.

Prostaglandin synthesis inhibitors have been extensively investigated in various murine cancers, and they usually show a beneficial effect (Bennett, 1982; 1986). In the NC tumour model the prostaglandin synthesis inhibitors indomethacin or flurbiprofen increase mouse survival and usually reduce tumour size. Survival is even longer when these nonsteroidal anti-inflammatory drugs are used with the cytotoxic drugs methotrexate and melphalan (Bennett *et al.*, 1982). The effects of the anti-inflammatory drugs seems likely to result from inhibition of prostaglandin synthesis rather than from other properties that the drugs may have (Flower, 1974), since administration of a $PGE_2$ analogue counteracted the effect of indomethacin (Bennett *et al.*, (1985). A similar effect might therefore be expected with other drugs that reduce prostaglandin formation, such as corticosteroids and mepacrine which can inhibit phospholipase activity and so depress the release of prostaglandin precursors (Vane *et al.*, 1982). This action may be particularly relevant to human cancers since prednisolone is frequently used in chemotherapy regimens. We have therefore studied the effects of prednisolone and mepacrine, alone and in combination with the cytotoxic drugs methotrexate and melphalan, in mice with NC tumours. Measurements were made of tumour weight and prostanoid content, the occurrence of metastases, and mouse survival.

### Materials and methods

The original NC tumour arose spontaneously in the mammary region of a WHT/Ht mouse (Hewitt *et al.*, 1976) and has since been passaged only in this strain. It has a high incidence of local lymphatic spread, recurrence in the scar following tumour excision, and metastasis mainly to the lungs and mediastinum.

On day 0, male or female WHT/Ht mice were injected s.c. into the left flank with $\sim 10^6$ NC carcinoma cells in a single-cell suspension from passaged tumours as described previously (Bennett *et al.*, 1979, 1982). All tumours were excised at 2 weeks and weighed. The study consisted of 6 separate experiments, each with 9–15 WHT/Ht mice/group. Drugs were given orally in 0.1 ml 50% syrup BP for 5 days (Monday to Friday) each week, and treatment with prednisolone or mepacrine was continued until death or the end of the experiment (day 121). The doses chosen are approximately the highest recommended amounts for man.

In experiments 1 and 2, prednisolone 500 $\mu g\,kg^{-1}$ (or vehicle for controls) were given daily from day 1 (the day after tumour inoculation) to female mice. In experiment 2, methotrexate 2 $mg\,kg^{-1}$ and melphalan 1.4 $mg\,kg^{-1}$ were given on days 15–17, 22–24, 29–31 with or without prednisolone.

In experiments 3 and 4, prednisolone 500 $\mu g\,kg^{-1}$ or vehicle were given daily from day 15 (the day after tumour excision) to female mice. In experiment 4, methotrexate and melphalan were given with prednisolone or vehicle as above.

Experiments 5 and 6 were with male mice given mepacrine hydrochloride 3.6 $mg\,kg^{-1}$ from day 1, alone or with methotrexate and melphalan as above. There was no rationale concerning the sex of the mice. We used what was available, but kept to the same sex in each study.

In studies where drug treatment began on day 1, some tumours were homogenised in acid-ethanol (Krebs solution acidified to $\sim$pH 3 with formic acid, and mixed with an equal volume of ethanol). Homogenisation in this solution gives 'basal' amounts of prostaglandins (Bennett *et al.*, 1973). After extraction (Unger *et al.*, 1971), PGE, 6-keto-$PGF_{1\alpha}$ and $TXB_2$ were measured by radioimmunoassay (Hennam *et al.*, 1974). The % cross-reactivities of the antibodies were as follows. PGE antibody (Miles Scientific): $PGE_2$ 100; $PGE_1$ 53; $PGF_{2\alpha}$ 10; $PGA_1$ 2.7; $PGF_{1\alpha}$ 2.6; $PGB_2$ 1.5; $PGA_2$ 1.4; $PGB_1$0.9. 6-Keto-$PGF_{1\alpha}$ antibody (Wellcome Research Laboratories): 6-keto-$PGF_{1\alpha}$ 100; $PGF_{2\alpha}$ 3.0; $PGE_2$ 0.1; $TXB_2$ 0.02. $TXB_2$ antibody: $TXB_2$ 100; $PGF_{2\alpha}$ 0.11; 6-keto-$PGF_{1\alpha}$ 0.01; $PGE_2$ <0.01. Intra- and inter-assay coefficients of variation were respectively 10–11% and 15–21% and the lower limits of detection were (pg per 100 $\mu$g): PGE 15.6; 6-keto-$PGF_{1\alpha}$ 12.5; $TXB_2$ 7.8. The tritiated compounds, obtained from Amersham International, had the following specific activities ($TBq\,mmol^{-1}$): $PGE_2$ 5.92; 6-keto-$PGF_{1\alpha}$ 5.55; $TXB_2$ 6.66. The bound and unbound compounds were separated by adding 1 ml of cold (4°C) ammonium sulphate/calcium sulphate (65% saturated ammonium sulphate solution pH 7.6 + calcium sulphate 1 g per 25 ml, maintained as an even suspension with a magnetic stirrer).

The mice were weighed twice weekly from at least 2 weeks prior to the start of the experiment up to death or day 121. Those with advanced carcinomatosis were killed humanely to prevent suffering (Bennett *et al.*, 1982). Survival time was measured from the day of tumour inoculation, and analysed by the method of Lee and Desu (1972). The incidences of recurrence in the excision scar, lymph nodes and or distant sites were noted at postmortem, and analysed by Fisher's exact test.

In another experiment, using 8 normal female mice/group, we investigated the effects of mepacrine 3.6 $mg\,kg^{-1}$ or prednisolone 500 $\mu g\,kg^{-1}$ on serum prostanoids. The mice were dosed daily for 2 days with drug or vehicle, and

Correspondence: A. Bennett.
Received 6 August 1986; and in revised form 13 November 1986.

anaesthetised with ether 2 h after the final dose. Blood was obtained by cardiac puncture and incubated at 37°C for 30 min to allow formation of $TXB_2$ during clotting. After centrifugation (1500 g, 4°C for 10 min), the serum was removed and stored at −20°C prior to radioimmunoassay of the unextracted samples for PGE, 6-keto-$PGF_{1\alpha}$ and $TXB_2$.

### Results

*Tumour weight, spread and host survival*

All transplanted tumours became palpable within 10 days. The weights of the tumours excised from mice treated from day 1 with prednisolone (experiments 1 and 2) were similar to the controls, being respectively 370 (240–440) mg $n=30$, and 300 (250–400) mg, $n=30$, $P>0.2$ (Mann–Whitney U-test). The weights of tumours from mice treated from day 1 with mepacrine (experiments 5 and 6) were also similar to controls, being respectively 240 (80–460) mg $n=45$, and 230 (120–380) mg $n=43$, $P>0.8$.

Neither prednisolone nor mepacrine improved mouse survival, regardless of whether treatment was started after the tumour was transplanted or excised. In fact, mice treated with prednisolone from day 1 (experiments 1 and 2) fared worse than the controls ($P<0.04$, Lee & Desu 1972; Table I). Cytotoxic chemotherapy alone (methotrexate and melphalan) improved survival, but this was not affected by combination with prednisolone or mepacrine (Table I). Figure 1 shows survival curves for mice given prednisolone 500 $\mu g\,kg^{-1}$ from day 1 (experiments 1 and 2).

**Figure 1** Treatment with the cytotoxic drugs methotrexate and melphalan (Cyto) increased the survival time of mice with resected NC tumours ($P=0.016$, Lee & Desu, 1972). Prednisolone 500 $\mu g\,kg^{-1}$ (Drug) given from day 1, the day after tumour transplantation, seemed to shorten the survival time (experiments 5 and 6), but it did not affect the response to the cytotoxic drugs (Drug + cyto).

The postmortem findings showed mainly similar incidences of recurrence in the excision scar, lymph nodes and lungs in the different treatment groups. The lack of effect on metastasis in mice given chemotherapy is not surprising, since they lived longer and had more time for tumour to spread, grow and eventually kill them.

*Tumour prostanoids*

The median tumour yield of 6-keto-$PGF_{1\alpha}$ (mice treated from day 1 with prednisolone) was 36% less than controls ($P<0.1$; experiment 1). In the mepacrine-treated mice (experiment 6) the median tumour PGE and $TXB_2$ were respectively 38% and 25% less then controls (both $P<0.1$). The other tumour prostanoid measurements were similar to controls (Table II). PGE was the predominant tumour prostanoid (medians for control males and females respectively 214 and 150 $ng\,g^{-1}$), and there was little $TXB_2$ (medians 12 and 5 $ng\,g^{-1}$; Table II).

*Serum prostanoids*

The serum contained more $TXB_2$ than PGE or 6-keto-$PGF_{1\alpha}$. In mice given prednisolone, the median amount of serum $TXB_2$ was 86% greater than control ($P=0.04$) whereas 6-keto-$PGF_{1\alpha}$ was 84% lower ($P=0.01$); the PGE was little changed (Table III). Mepacrine-treated mice also had less 6-keto-$PGF_{1\alpha}$ (median 74% lower than control, $P=0.04$), but the other prostanoids were little affected.

### Discussion

As reported previously (Bennett *et al.*, 1982; 1985), chemotherapy with methotrexate and melphalan increased the survival of mice with resected NC tumours. We started these experiments in the expectation that prednisolone and mepacrine would act as phospholipase inhibitors, thereby lowering prostaglandin production and mimicking the beneficial effect of cyclooxygenase inhibitors in mice with NC tumours (Bennett *et al.*, 1979; 1982). Lynch *et al.* (1978) found that indomethacin, aspirin or hydrocortisone increased the survival of mice with methylcholanthrene-induced tumours, although only the nonsteroids significantly reduced the tumour size. Inhibition of prostaglandin synthesis appears to explain the antitumour effect of indomethacin on the NC cancer, since a stable $PGE_2$ analogue counteracted the increase in survival (Bennett *et al.*, 1985). However, in the present experiments prednisolone or mepacrine had little or no effect on the cancer or its response to the cytotoxic drugs. An explanation for this lack of anticancer activity may be that although the doses/kg of prednisolone and mepacrine are near the maximum used in man, they caused at most a weak inhibition of prostanoid formation by the mouse tumours. Furthermore, although they reduced the amount of 6-keto-$PGF_{1\alpha}$ in serum from normal mice, the PGE seemed

**Table I** Mouse survival

| *Drug* | *Day treated* | *Controls* | *Drug-treated* | *CT* | *Drug + CT* |
|---|---|---|---|---|---|
| Prednisolone 500 $\mu g\,kg^{-1}$ | D1 | 39 (36–43) $n=19$ | 37 (33–38) $n=19$ | 45 (42–46)a $n=10$ | 48 (42–52) $n=10$ |
| Prednisolone 500 $\mu g\,kg^{-1}$ | D15 | 37 (35–40) $n=19$ | 38 (35–44) $n=20$ | 51 (51–55)b $n=10$ | 49 (47–53) $n=10$ |
| Mepacrine 3.6 $mg\,kg^{-1}$ | D1 | 41 (38–50) $n=23$ | 39 (34–49) $n=23$ | 52 (45–55)c $n=17$ | 51 (47–64) $n=18$ |

Prednisolone given from day 1 (D1, experiments 1 and 2) or day 15 (D15, experiments 3 and 4) or mepacrine from D1 (experiments 5 and 6) had little or no effect on mouse survival (Lee & Desu, 1972). The results are days, shown as medians with semiquartile ranges in parentheses. Survival was lengthened by chemotherapy (CT) with methotrexate and melphalan, but addition of prednisolone or mepacrine did not affect the response to CT.

*P* values: a = 0.02 (experiment 2); b < 0.005 (experiment 4); c < 0.0005 (experiments 5 and 6).

**Table II** Radioimmunoassay of tumour prostanoids

| *Treatment* | *Sex* | *PGE* | *6-Keto-PGF$_{1\alpha}$* | *TXB$_2$* |
|---|---|---|---|---|
| Controls | F | 150 (103–193) $n=10$ | 87 (62–154) $n=10$ | 5 (4–8) $n=10$ |
| 500 $\mu$g kg$^{-1}$ prednisolone | F | 102 (94–141) $n=10$ | 56 (62–68) $n=10$ | 4 (3–5) $n=10$ |
| Controls | M | 214 (120–278) $n=19$ | 55 (39–88) $n=20$ | 12 (9–14) $n=20$ |
| 3.6 mg kg$^{-1}$ mepacrine hydrochloride | M | 133 (88–198) $n=19$ | 47 (32–94) $n=20$ | 9 (8–11) $n=20$ |

In all 12 cases (experiments 1 to 6) the tumours from drug-treated mice yielded smaller median amounts of prostanoids when homogenised in acid-ethanol, but the $P$ values were $<0.1$ compared to controls in only 3 groups (prednisolone, 6-keto-PGF$_{1\alpha}$; mepacrine PGE, TXB$_2$). The results are ng g$^{-1}$ wet issue, shown as medians with semiquartile ranges in parentheses.

**Table III** Serum prostanoids

| | *PGE* | *6-keto-PGF$_{1\alpha}$* | *TXB$_2$* |
|---|---|---|---|
| Control | 9 (8–11) | 25 (23–27) | 168 (160–175) |
| Prednisolone 500 $\mu$g kg$^{-1}$ | 9 (8–9) | 4 (4–5)[b] | 312 (293–390)[a] |
| Mepacrine 3.6 mg kg$^{-1}$ | 11 (10–11) | 9( 6–22)[a] | 219 (127–300) |

Prednisolone-treated normal female mice had less 6-keto-PGF$_{1\alpha}$ and more TXB$_2$ in the serum samples. The same trend occurred with mepacrine. [a]$P=0.04$ [b]$P=0.01$ (Mann–Whitney U-test). Values are ng ml$^{-1}$ shown as medians with semiquartile ranges in parentheses. In all groups $n=8$, except for mepacrine and 6-keto-PGF$_{1\alpha}$ where $n=7$.

to be unaffected and the amount of TXB$_2$ was actually greater. Rittenhouse-Simmons and Deykin (1981) considered that failure of platelets to synthesise new protein explains why their prostanoid formation is not blocked by corticosteroids. However, this may not be correct since there is some evidence that platelets can incorporate amino acids into protein (Shaw *et al.*, 1984).

The possibility that the doses were too low to affect extravascular prostanoid formation prompted us to study prednisolone 0.5–15 mg kg$^{-1}$ or mepacrine 3.6–28.8 mg kg$^{-1}$ given orally to normal mice for 3 days. These doses had little or no effect on the amounts of PGE, 6-keto-PGF$_{1\alpha}$ or TXB$_2$ extracted from intestine homogenised in acid-ethanol (unpublished). Although this seems to be contrary to current thinking (Vane *et al.*, 1982), various groups have reported that *in vivo* or with intact cells *in vitro* corticosteroids did not affect prostaglandin synthesis. For example there was no effect of dexamethasone on the amount of peritoneal prostaglandins in rats (Deraedt *et al.*, 1980), or on prostaglandin formation by rat polymorphonuclear leucocytes (Dray *et al.*, 1980). In patients given 6$\alpha$-methyl-prednisolone the concentrations of prostanoids in synovial effusions were variably affected: 6-keto-PGF$_{1\alpha}$ fell by 35% and PGF$_{2\alpha}$ increased by 30%, while PGE$_2$ and TXB$_2$ were unchanged (Bombardieri *et al.*, 1981). The latter results, and our findings with mouse serum, suggest that the effects of prednisolone and mepacrine are more like those expected of a PGI$_2$ synthetase inhibitor than a phospholipase A2 inhibitor, there being in general less 6-keto-PGF$_{1\alpha}$ and more TXB$_2$. Clearly inhibition of prostanoid synthesis by these drugs *in vivo* does not seem to be a universal occurrence.

According to Honn *et al.* (1981, 1983), increasing the prostacyclin:thromboxane ratio in blood decreases metastasis by reducing platelet aggregation. Since this ratio decreased with prednisolone or mepacrine, we might have expected the drugs to worsen the cancer. Measurement of blood prostanoids is fraught with difficulties, and furthermore we used serum so that some of the 6-keto-PGF$_{1\alpha}$ may have originated from PGI$_2$ formed during clotting. Nevertheless, our findings are weak evidence against Honn's hypothesis. They are consistent with our finding that the thromboxane synthetase inhibitor dazmegrel reduced mouse serum TXB$_2$ and increased the 6-keto-PGF$_{1\alpha}$, but had no effect on the survival of mice bearing NC tumours (Stamford *et al.*, 1986). Similarly, recent work in breast cancer patients demonstrated that the TXB$_2$:6-keto-PGF$_{1\alpha}$ ratio in the systemic circulation is not an indicator of malignancy or metastasis (Nigam *et al.*, 1985).

We thank the CRC and MRC for support.

### References

BENNETT, A. (1982). Prostaglandins and inhibitors of their synthesis in cancer growth and spread. In *Endocrinology of Cancer*, Rose, D.P. (ed) Vol. 3, p. 113. CRC Press Inc.: Boca Raton.

BENNETT, A. (1986). Prostaglandins and cancer. In *CRC Handbook of Eicosanoids and Related Lipids*, Willis *et al.* (eds) (in press). CRC Press Inc.: Boca Raton.

BENNETT, A., BERSTOCK, D.A. & CARROLL, M.A. (1982). Increased survival of cancer-bearing mice treated with inhibitors of prostaglandin synthesis alone or with chemotherapy. *Br. J. Cancer*, **45**, 762.

BENNETT, A., CARROLL, M.A., MELHUISH, P.B. & STAMFORD, I.F. (1985). Treatment of mouse carcinoma *in vivo* with a prostaglandin E$_2$ analogue and indomethacin. *Br. J. Cancer*, **52**, 245.

BENNETT, A., HOUGHTON, J., LEAPER, D.J. & STAMFORD, I.F. (1979). Cancer growth, response to treatment and survival time in mice: Beneficial effect of the prostaglandin synthesis inhibitor flurbiprofen. *Prostaglandins*, **17**, 179.

BENNETT, A., STAMFORD, I.F. & UNGER, W.G. (1973). Prostaglandin $E_2$ and gastric acid section in man. *J. Physiol.*, **229**, 349.

BOMBARDIERI, S., CATTANI, P., LIABETTONI, G. & 5 others (1981). The synovial prostaglandins system in chronic inflammatory arthritis: differential effects of steroidal and nonsteroidal anti-inflammatory drugs. *Br. J. Pharmacol.*, **73**, 893.

DERAEDT, R., JOUQUEY, S., DELEVALLEE, F. & FLAHAUT, M. (1980). Release of prostaglandins E and F in an algogenic reaction and its inhibition. *Eur. J. Pharmacol.*, **61**, 17.

DRAY, F., McCALL, E. & YOULTEN, L.J.F. (1980). Failure of anti-inflammatory steroids to inhibit prostaglandin production by rat polymorphonuclear leucocytes. *Br. J. Pharmacol.*, **68**, 199.

FLOWER, R.J. (1974). Drugs which inhibit prostaglandin biosynthesis. *Pharmacol. Rev.*, **26**, 33.

HENNAM, J.P., JOHNSON, D.A., NEWTON, J.R. & COLLINS, W.P. (1974). Radioimmunoassay of prostaglandin $F_{2\alpha}$ in peripheral venous plasma from men and women. *Prostaglandins*, **5**, 531.

HEWITT, H.B., BLAKE, E.R. & WALDER, A.S. (1976). A critique of the evidence for active host defence against cancer, based on personal studies of 27 murine tumours of spontaneous origin. *Br. J. Cancer*, **33**, 241.

HONN, K.V., BUSSE, W.D. & SLOANE, B.F. (1983). Prostacyclin and thromboxanes, Implications for their role in tumor cell metastasis. *Biochem. Pharmacol.*, **32**, 1.

HONN, K.V., CICONE, B. & SKOFF, A. (1981). Prostacyclin a potent anti-metastatic agent. *Science*, **212**, 1270.

LEE, E. & DESU, M. (1972). A computer programme for comparing K samples with right-censored data. *Comp. Prog. Biomed.*, **2**, 315.

LYNCH, N.R., CASTES, M., ASTOIN, M. & SALOMON, J.C. (1978). Mechanism of inhibition of tumour growth by aspirin and indomethacin. *Br. J. Cancer*, **38**, 503.

NIGAM, S., BECKER, R., ROSENDAHL, U. & 4 others (1985). The concentrations of 6-keto-$PGF_{1\alpha}$ and $TXB_2$ in plasma samples from patients with benign and malignant tumours of the breast. *Prostaglandins*, **29**, 513.

RITTENHOUSE-SIMMONS, S. & DEYKIN, D. (1981). Release and metabolism of arachidonate in human platelets. In *Platelets in Biology and Pathology* 2, Gordon, J.L. (ed) p. 349. Elsevier: North Holland.

SHAW, T., CHESTERMAN, C.N. & MORGAN, F.J. (1984). *In vitro* synthesis of low molecular weight proteins in human platelets: absence of labelled release products. *Thrombosis Res.*, **36**, 619.

STAMFORD, I.F., MELHUISH, P.B., CARROLL, M.A., CORRIGAN, C., PATEL, S. & BENNETT, A. (1986). Survival of mice with NC carcinoma is unchanged by drugs that are thought to inhibit thromboxane synthesis or increase prostacyclin formation. *Br. J. Cancer*, **54**, 257.

UNGER, W.G., STAMFORD, I.F. & BENNETT, A. (1971), Extraction of prostaglandins from human blood. *Nature*, **233**, 336.

VANE, J.R., FLOWER, R.J. & SALMON, J.A. (1982). Inhibitors of arachidonic acid metabolism, with especial reference to the aspirin-like drugs. In *Prostaglandins and Cancer: First International Conference*, Powles, T.J. *et al.* (eds) p. 21. Alan R. Liss: New York.